# Alcohol Abuse and Smoking: Dual Recoveries

**Published:** 1996

**Authors:** Linda C. Sobell, Mark B. Sobell

**Affiliations:** Linda C. Sobell, Ph.D., is a professor at Nova Southeastern University, Ft. Lauderdale, Florida, and a senior scientist at the Addiction Research Foundation, Toronto, Ontario, Canada. Mark B. Sobell, Ph.D., is a professor at Nova Southeastern University, Ft. Lauderdale, Florida, and a senior scientist at the Addiction Research Foundation, Toronto, Ontario, Canada

**Keywords:** AOD abuse, cigarette, smoking, AODD (alcohol and other drug use disorder) recovery, AODD relapse, cessation of AODU (alcohol and other drug use), relapse prevention, treatment

## Abstract

Between 80 and 95 percent of all alcohol abusers smoke cigarettes, leading to enormous public health problems. Little is known, however, about how changes in drinking and/or smoking affect recovery from both of these problems. A long-term study of alcohol abusers who also had been smokers at some point in their lives found that most of these people had recovered from both alcohol abuse and smoking and that these recoveries were very stable. Continued smoking, however, generally was associated with an increased risk for relapse to alcohol abuse.

Based on figures from national household surveys, researchers estimate that about 70 percent of the adult population consume some alcohol and that approximately 10 to 20 percent are alcohol abusers (reviewed in Sobell and Sobell 1993). (In this article, the term “alcohol abuser” refers to all people whose alcohol consumption has led to medical, psychological, or social problems and therefore encompasses the diagnoses of alcohol abuse and alcohol dependence as defined in the American Psychiatric Association’s *Diagnostic and Statistical Manual of Mental Disorders, Fourth Edition*.) Moreover, about 30 percent of the adult population smoke cigarettes, although smoking rates have declined over the past decade (Fiore et al. 1989). Research has shown that alcohol and nicotine use are strongly linked (for a recent summary of the literature, see Fertig and Allen 1995) and that 80 to 95 percent of all alcohol abusers smoke cigarettes (for review, see Toneatto et al. 1995).

The public health problems and costs associated with the use and abuse of these two widely available and legal drugs are monumental. For example, alcohol abuse is a major cause of morbidity and mortality in North America (Brady 1995; Institute of Medicine 1990) and is the leading cause of alcoholic cirrhosis. Similarly, the fact that “tobacco smoking accounts for more morbidity and mortality in America today than use of all other psychoactive drugs combined” (Brady 1995, p. 123) attests to its costs and consequences. Cigarette smoking is the single most important cause of cancer (U.S. Department of Health and Human Services [USDHHS] 1988). Although the costs to society of abusing either alcohol or tobacco are substantial (National Institute on Alcohol Abuse and Alcoholism 1993; USDHHS 1988), the cost of their combined use is even greater (Brady 1995). For example, some researchers have hypothesized that the interaction of alcohol and nicotine can induce laryngeal cancer and that the risk for this cancer in alcohol abusers who smoke is about 50 percent higher than the sum of the two independent risks from smoking or drinking alcohol (see Brady 1995).

Despite the frequent co-occurrence of alcohol and tobacco use, only a few studies have explored the relationship between recovery from smoking (Brady 1995) and recovery from alcohol-related problems (Sobell et al. 1995). Most available information about the effects of alcohol-tobacco interactions derives from analyses of clients in alcohol treatment programs or from short-term followup studies of such programs. Although evidence exists that concurrent treatment for smoking does not decrease recovery rates from drinking (Bien and Burge 1990; Bobo et al. 1986), little is known about how changes in drinking affect attempts to stop smoking and vice versa. This article reviews the results of two studies that shed some light on this relationship. These studies have focused on people who recovered from alcohol abuse or alcohol dependence without formal help or treatment and who also stopped smoking.

## Study 1

The Canadian National Alcohol and Other Drugs Survey (NADS) (Statistics Canada 1990) was based on telephone interviews with 11,634 randomly selected people that evaluated the respondents’ alcohol and other drug (AOD) use. The interview questions identified and classified recovered alcohol abusers according to their drinking statuses at the time of the interviews. The recovered respondents fell into two groups: those who had become abstinent (62 percent of all recovered alcohol abusers) and those who had returned to moderate drinking (38 percent of the recovered abusers). More specifically, the study identified 302 recovered respondents who had been abstinent for 1 or more years when interviewed (i.e., resolved abstinent respondents) and 144 respondents who reported that they had once experienced alcohol problems but had reduced their alcohol consumption to a moderate level for at least 1 year (i.e., resolved nonabstinent respondents). Of these 446 recovered subjects, 77.5 percent had received no formal help or treatment during their recoveries. (For more information on the subjects’ drinking recoveries, drinking criteria, and personal characteristics, see Sobell et al. 1996.)

The NADS also included questions about the subjects’ past and present smoking behavior. When the smoking histories of the recovered alcohol abusers were compared, the findings indicated that more of the resolved abstinent subjects (85.3 percent) had smoked at some time in their lives compared with the resolved nonabstinent subjects (68.5 percent) (Sobell et al. 1995). This finding was consistent with previous analyses suggesting that resolved abstinent subjects have more severe histories of alcohol-related problems (i.e., are more likely to have been alcohol dependent**)** than resolved nonabstinent subjects (Sobell et al. 1992).

In addition, the study examined the correlation between smoking status at the time of the interview and the type (i.e., abstinent versus nonabstinent) and length (i.e., from 1 to 5 years versus more than 5 years) of recovery from alcohol abuse (table 1). These analyses detected no significant differences in smoking cessation rates between short-and long-term resolved nonabstinent respondents. In contrast, the number of resolved abstinent respondents who continued smoking was significantly higher among those with short-term recoveries than among those with long-term recoveries (for more information, see Sobell et al. 1995). In view of the suggestion discussed previously that the resolved abstinent subjects may have had more severe alcohol-related problems than did the resolved nonabstinent subjects, one possible interpretation of this finding is that people with more severe drinking problems may have greater difficulty sustaining their recoveries over the long term if they continue to smoke.

## Study 2

The correlation between recovery from alcohol abuse and smoking cessation also was examined in a long-term study of alcohol abusers who recovered without receiving formal treatment (Sobell et al. 1992, 1993). The 120 participants, who had been recruited through media advertisements, were classified into three groups: resolved abstinent subjects, resolved nonabstinent subjects, and resolved abstinent treatment subjects. The 71 resolved abstinent subjects, all of whom had been alcohol dependent according to the criteria of the American Psychiatric Association’s *Diagnostic and Statistical Manual of Mental Disorders, Third Edition, Revised* (DSM–III–R), had recovered from problem drinking through abstinence for 3 or more years when interviewed. In contrast, the 21 resolved nonabstinent subjects, 90.5 percent of whom had met the DSM–III–R criteria for alcohol dependence, were nonproblem drinkers (i.e., their alcohol consumption met the criteria for nonhazardous use and did not lead to any alcohol-related problems) for at least 3 years when interviewed. Finally, the study included 28 subjects with diagnoses of alcohol dependence according to the DSM–III–R criteria who had been abstinent for at least 1 year and who had received some sort of treatment in the past but asserted that their abstinence was independent of this treatment (i.e., resolved abstinent treatment subjects). Because the three groups did not differ significantly in their reasons for initiating recovery or in the factors that helped them maintain their recovery, all subjects are combined in the following discussion.

Five years after the initial interview, the subjects were reinterviewed to assess the stability of their recoveries. Of the 113 subjects still alive at that time, 109 were reinterviewed and corresponding information was available for two additional subjects, allowing the evaluation of 111 of the original study participants.

At the initial interview, 110 of the 120 subjects reported that they had been smokers at some time in their lives. Of these, 33 percent were still smoking when first interviewed, whereas 67 percent had recovered from both alcohol abuse and smoking. The timing of drinking and smoking cessation varied among the subjects: A total of 49 percent of the subjects recovered from alcohol abuse before they stopped smoking, 40 percent stopped smoking first, and 11 percent stopped drinking and smoking concurrently (i.e., within 1 year). Of those who first quit smoking, 30 percent did so before they began to abuse alcohol. Additional analyses demonstrated that those subjects who had more severe alcohol- and smoking-related problems tended to recover from their drinking problems before stopping smoking (Sobell et al. 1992, 1993).

At reinterviews, 57 subjects who had experienced concurrent alcohol- and smoking-related problems (i.e., excluding those who had quit smoking before they developed alcohol-related problems) were asked to rate which problem was more difficult to resolve. Consistent with previous research (Kozlowski et al. 1989), 40 percent of the subjects considered giving up smoking more difficult, 28 percent rated resolving a drinking problem as more difficult, and 32 percent reported no difference. Furthermore, subjects who had achieved dual recoveries reported similar numbers of previous attempts to quit smoking (2.2 attempts) or resolve their drinking problems (2.5 attempts).

### Stability of Long-Term Recoveries

Because the study included followup interviews with the same subjects 5 years after the initial interviews (i.e., the study was longitudinal), it allowed the researchers to compare the subjects’ relapse rates to drinking and smoking. These analyses demonstrated exceptionally low relapse rates, indicating that long-term recoveries from both smoking and alcohol abuse were very stable. Of the 111 subjects for whom followup information was available, only 17 (15 percent) had relapsed to alcohol abuse over the 5-year interval (Sobell et al. 1995).

Relapse rates for smoking were even lower: Of the subjects who had stopped smoking by the first interview and were available for followup, only 6 percent relapsed between interviews. The high stability of smoking patterns also is underscored by the following findings: Overall, 97 of the 109 subjects for whom smoking data were available (89 percent) maintained their smoking status during the followup period, including 10 who never smoked, 28 who remained smokers (8 smokers quit between interviews), and 59 who remained quitters. Thus, at both interviews, two-thirds of the recovered alcohol abusers who had ever smoked also had stopped smoking (Sobell et al. 1995).

### Continued Smoking Increases Risk of Relapse to Drinking

Recovered alcohol abusers who continued to smoke cigarettes had a significantly higher risk of relapse to drinking than recovered alcohol abusers who also had stopped smoking (table 2) (Sobell et al. 1995). At the second interview, 28.1 percent of those subjects who were still smoking had relapsed to drinking, whereas only 7.5 percent of the subjects who had quit smoking had relapsed to drinking in the 5-year period between the interviews. Similarly, only 10 percent of the subjects who had never smoked had relapsed to drinking by the second interview. Although the numbers of subjects studied are small, these findings suggest that continued smoking increases the risk of relapse for recovered alcohol abusers. Alternatively, another unrelated factor may be responsible for both continued smoking and the relapse to alcohol abuse.

## Implications for Treatment and Relapse Prevention of Alcohol Abusers Who Smoke

The frequent co-occurrence of alcohol use and smoking suggests that similar cues (e.g., bars and restaurants) can induce both behaviors. Because these cues would include those associated with the very act of consumption (i.e., smoking or drinking alcohol), one would expect that attempts to stop using one drug while continuing to use the other would be more difficult than quitting both drugs simultaneously. Contrary to that assumption, however, concurrent cessation of smoking and drinking occurred infrequently among the subjects in the two studies described here who had recovered from alcohol abuse without formal treatment.

One interesting finding was that more subjects with severe, as opposed to less severe, alcohol-related problems stopped smoking after they resolved their drinking problems. Because the subjects with more severe alcohol-related problems also had significantly longer histories of smoking (Sobell et al. 1992) and thus may have been more dependent on nicotine, this finding may mean that recovering from severe alcohol-related problems is easier than recovering from long-term smoking.

Both studies demonstrated a complex relationship between drinking and smoking. Although continued smoking generally was associated with a higher relapse risk for alcohol abuse, some successfully recovered alcohol abusers maintained stable levels of moderate drinking while continuing to smoke, whereas other moderate drinkers also had stopped smoking. Thus, it appears that continued smoking does not always result in alcohol abuse relapse and that continued moderate drinking does not invariably lead to smoking relapse. These observations underscore the need for further research on the advantages or limitations of recovering from both problems simultaneously as well as on the contents of treatment for both alcohol abuse and smoking. To date, no treatment trials have examined whether concurrent or sequential treatments for alcohol abuse and smoking would be more effective.

In addition, future studies should evaluate the influence of client characteristics (e.g., severity of alcohol-related problems, strength of association between smoking and drinking, and/or degree of compulsive behavior) on treatment outcomes. Such analyses could lead to the development of criteria for matching individual patients to various treatment approaches. Given the large numbers and the diversity of people who both smoke and abuse alcohol, it seems highly unlikely that any single treatment formulation will be suitable for all clients. Consequently, clinicians should probably take into account individual differences among alcoholic smoking patients in order to develop effective treatment strategies.

## Figures and Tables

**Table 1:** Smoking Status at the Time of Interview for Recovered Alcohol Abusers by Type and Length of Recovery^1^

Smoking Status	Recovery Group by Recovery Length			

	Resolved Abstinent (%)		Resolved Nonabstinent (%)	
	
	1–5 Years (*n* = 92)	> 5 Years (*n* = 140)	1–5 Years (*n* = 39)	> 5 Years (*n* = 50)
Still Smoking	75.8	54.1	41.5	36.2
Stopped Smoking	24.2	45.9	58.5	63.8

1 Data from respondents of the 1989 Canadian National Alcohol and Other Drugs Survey who were more than 20 years of age (*n* = 10,796). Percentages reflect weighted data (i.e., data have been adjusted to account for various aspects of the sampling design), whereas sample sizes (i.e., *n*) are unweighted data.

**Table 2:** Relationship of Smoking Status and Relapse to Drinking at Interview 2 for 109 Subjects Recovered From Alcohol Problems at Interview 1

Smoking Status at Interview 2	Drinking Status at Interview 2		Relapse Rate (%) *n_2_*/*(n_1_+n_2_)*

	Still Recovered *n_1_*	Relapsed *n_2_*	
Current Smoker	23	9	28.1
Quit Smoking	62	5	7.5
Never Smoked	9	1	10.0
